# Transcriptomic profiles of retinal ganglion cells are defined by the magnitude of intraocular pressure elevation in adult mice

**DOI:** 10.1038/s41598-019-39141-1

**Published:** 2019-02-22

**Authors:** Yong H. Park, Joshua D. Snook, Edwin J. Ostrin, Sangbae Kim, Rui Chen, Benjamin J. Frankfort

**Affiliations:** 1 Department of Ophthalmology, Baylor College of Medicine, Houston, Texas 77030 United States; 2 Pulmonary Medicine, The University of Texas MD Anderson Cancer Center, Houston, TX 77030 United States; 3 Department of Molecular and Human Genetics, Baylor College of Medicine, Houston, Texas 77030 United States; 4 Human Genome Sequencing Center, Baylor College of Medicine, Houston, Texas 77030 United States; 5 Department of Neuroscience, Baylor College of Medicine, Houston, Texas 77030 United States

## Abstract

Elevated intraocular pressure (IOP) is the major risk factor for glaucoma, a sight threatening disease of retinal ganglion cells (RGCs) and their axons. Despite the central importance of IOP, details of the impact of IOP elevation on RGC gene expression remain elusive. We developed a 4-step immunopanning protocol to extract adult mouse RGCs with high fidelity and used it to isolate RGCs from wild type mice exposed to 2 weeks of IOP elevation generated by the microbead model. IOP was elevated to 2 distinct levels which were defined as Mild (IOP increase >1 mmHg and <4 mmHg) and Moderate (IOP increase ≥4 mmHg). RNA sequencing was used to compare the transcriptional environment at each IOP level. Differentially expressed genes were markedly different between the 2 groups, and pathway analysis revealed frequently opposed responses between the IOP levels. These results suggest that the magnitude of IOP elevation has a critical impact on RGC transcriptional changes. Furthermore, it is possible that IOP-based set points exist within RGCs to impact the direction of transcriptional change. It is possible that this improved understanding of changes in RGC gene expression can ultimately lead to novel diagnostics and therapeutics for glaucoma.

## Introduction

Glaucoma is a heterogenous group of optic neuropathies hallmarked by cupping of the optic nerve head and progressive death of retinal ganglion cells (RGCs), which results in a decrease and subsequent loss of vision^1^. Worldwide, glaucoma is a major cause of irreversible blindness, affecting over 70 million people^2^. This number includes over 3 million people in the United States, most of whom have primary open angle glaucoma (POAG)^2,3^. In glaucoma, the progression from visual dysfunction to overt vision loss is chronic and subtle, which creates multiple challenges in diagnosis, and in many cases profound and permanent vision loss has already occurred at the time of diagnosis^4,5^. Both human and animal studies suggest that it is very likely that both RGC function and retinal processing become abnormal prior to RGC death^6–14^, but the specific pathophysiological mechanisms that initially injure RGCs are poorly understood.

Elevated intraocular pressure (IOP) is the most important risk factor associated with glaucoma and correlates with the onset and progression of disease^4,5,15^. While reducing IOP can slow or arrest the progression of RGC loss, elevation of IOP alone does not determine if patients will develop glaucoma, and many patients with statistically elevated IOP do not develop glaucoma at all^16^. To try to better understand this complex relationship among IOP, RGCs, and glaucoma, a variety of animal models designed to study the impact of IOP on the retina have been developed^17–20^. In mice, IOP is commonly increased by the impediment of the aqueous outflow at the trabecular meshwork or episcleral vein, either by microbead injection^8,21,22^, laser cauterization^23,24^, hypertonic saline injection^25^, or spontaneous ciliary exfoliation^26,27^. These animal models have all demonstrated anatomic deficits similar to those seen in patients with glaucoma, and many have shown functional deficits as well^8–10,24,28–30^.

To try to understand the molecular causes of RGC dysfunction following IOP elevation, several transcriptomic studies of rodents with increased IOP have been performed^31–38^. While generally successful at identifying differentially expressed genes (DEGs) and pathways, these studies have important limitations. First, the IOPs in these animal models were commonly 100–200% of normal^18^, whereas the IOP increases generally seen in POAG are much less profound^5^. Second, in genetic spontaneous models (i.e. DBA2/J mice) the time point at which IOP elevation occurred is often difficult to define^26,31^. Third, studies in induced and genetic models have been carried out in a variety of genetic backgrounds and ages^31–33,39^. Fourth, many studies did not obtain pure and viable RGC populations due isolation techniques that utilize whole retina samples^32,33,36^, laser capture microdissections^38^ and non-specific antigen isolation^40^. Fifth, some studies were performed using older techniques such as microarrays^32–34^, which prevent the open-ended discovery of abnormal RNA transcription. Taken together, we are left with a muddled picture of the molecular impact of elevated IOP on RGCs in experimental mouse models.

In this study, we overcome the above limitations by using a variation of the microbead injection model to induce less dramatic IOP increases in a pure wild type mouse strain (C57BL/6J) at a specific age (6 weeks) and for a specific period of time (2 weeks). We also applied strict IOP criteria to define two IOP elevation levels (Mild and Moderate) which are similar to those seen in POAG. We then used a modified 4-step immunopanning technique that produced major improvements over other techniques to produce RGCs of high yield, purity, and viability. Finally, we applied Next-Generation Sequencing (NGS) by utilizing RNA-Sequenced messenger RNA (mRNA) cDNA constructs to determine differential transcriptome changes that occurred among the experimental IOP and control groups. Transcriptome and pathway analyses demonstrated two distinct patterns of change which were dependent on the magnitude of IOP increase, suggesting a complex response of RGCs to IOP challenges.

## Results

### Isolation of Adult RGCs from Mouse Utilizing a Modified 4-step Immunopanning Technique

**Figure 1:**
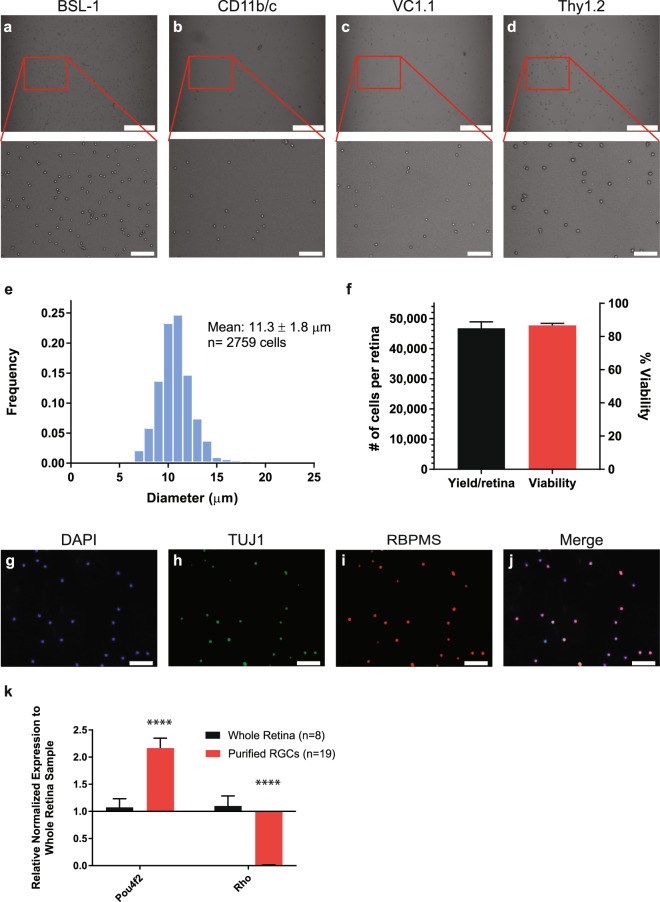
Characterization of Purified RGCs Isolated from Adult Mice. (**a**–**d**) Brightfield images (10x) were taken following each panning step during the isolation of adult RGCs from mice: (**a**) BSL-1 (fibroblasts, endothelial cells, macrophages and microglia), (**b**) CD11b/c (macrophages and microglia), (**c**) VC1.1 (amacrine cells), and (**d**) Thy1.2 (RGCs; scale bar = 250 µm). As expected, Thy1.2^+^ cells (inset, **d**) were larger than BSL-1^+^ (inset, **a**), CD11b/c^+^ (inset, **b**), and VC1.1^+^ (inset, **c**) cells (scale bar = 50 µm). (**e**) Bar graph showing the distribution of the diameter (µm) of the Thy1.2^+^ cells (n = 2,759 cells). The average cell diameter was 11.3 ± 1.8 µm. (**f**) 4-step immunopanning of RGCs average yield of 46,718 ± 2,176 cells per retina with an 86.7 ± 1.2% viability per isolation (n = 21). Immunostaining of the isolated cells against RGC Markers, TUJ1 (**h**) and RBPMS (**i**), as well as a nuclear stain, DAPI (**g**; scale bar = 50 µm). Merged image (**j**) identified 88.1 ± 4.2% of DAPI positive cells to be RBPMS positive. (**k**) Semi-quantitative q-PCR analysis identified a significant (p < 0.0001) increase in expression of *Pou4f2* (RGC marker) and significant decrease (p < 0.0001) in the expression of Rhodopsin (*Rho*; Photoreceptor gene marker) in the purified RGCs samples compared to the whole retina.

We modified a well-established 2-step RGC immunopanning technique^41,42^ by adding two additional negative panning steps, which resulted in a 4-step immunopanning protocol (Methods). Using 8 week old mouse retinas, this technique successfully removed macrophages, microglia, fibroblasts, endothelial cells, and amacrine cells, while allowing the retention of RGCs. As expected, the diameter of the isolated RGCs was consistently larger than other cell types (Fig. 1a–d). RGCs varied in diameter from a range of 7 to 17 µm (n = 2,759 cells) and had an average diameter of 11.3 ± 1.8 µm (Fig. 1e), consistent with expectations^43^. RGCs were recovered with high yield and viability, (average yield = 46,718 ± 2,176 cells per retina; average viability = 86.7 ± 1.2%; n = 21; Fig. 1f). We confirmed our enriched RGC population with immunocytochemistry of isolated Thy1.2^+^ cells and found that 88.1 ± 4.2% of cells stained for RBPMS, a well-established RGC marker^44,45^ (Fig. 1g–j). Additional confirmation of RGC purity was confirmed comparing isolated purified RGC samples (n = 19) to whole retina samples (n = 8) via by quantitative polymerase chain reaction (qPCR) of *Pou4f2* (RGC marker) and *Rho* (rod photoreceptor marker). *Pou4f2* showed a 2.2 ± 0.2-fold increase in gene expression (P-value < 0.0001), and *Rho* showed a 57.0 ± 0.0-fold decrease in gene expression (P-value < 0.0001) in purified RGC samples (Fig. 1k). Together, these data indicate that our modified 4-step immunopanning technique is successful in isolating purified RGCs with low cell contaminants.

### Transcriptomic Profiling Analysis of RGCs Following Intraocular Pressure Elevation at Two Distinct Levels

**Figure 2:**
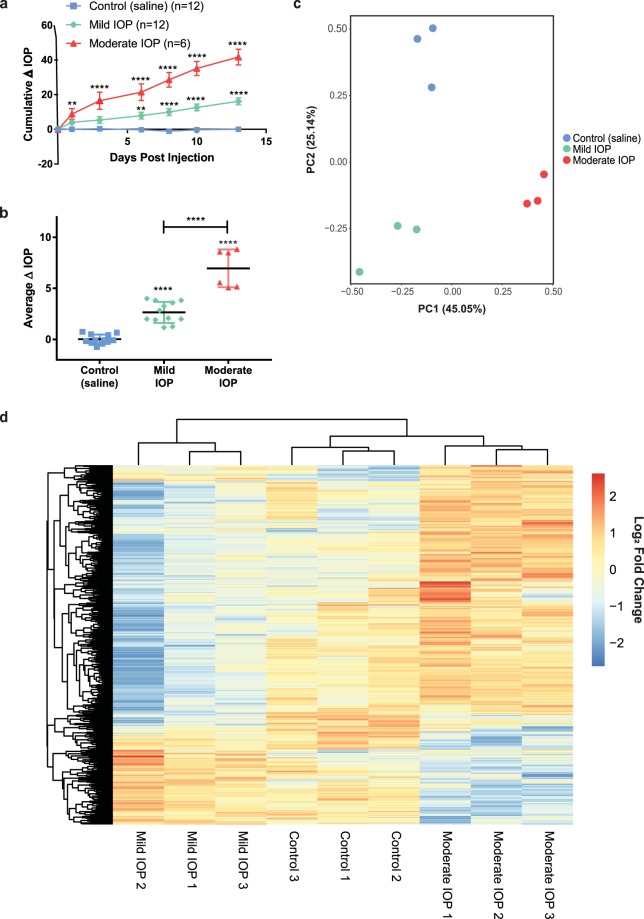
Transcriptomic Profiling Analysis of RGCs Following Mild and Moderate IOP Elevation. (**a**) Cumulative change in IOP for eyes exposed to Mild IOP (green), Moderate IOP (red), and Control (blue) conditions over a 2 week period. (**b**) Mean change in IOP per group. IOP levels were significantly increased after Mild (2.7 ± 0.3 mmHg) and Moderate (7.0 ± 0.8 mmHg) IOP increase when compared to Controls (0.0 ± 0.1 mmHg). For (**a**,**b**), **P < 0.01 and ****P < 0.0001. (**c**) PCA plot of Control, Mild IOP, and Moderate IOP in triplicate. (**d**) Hierarchical dendrogram clustering/heatmap of Control, Mild IOP, Moderate IOP samples’ gene expression with FPKM > 16.

To determine the transcriptomic profile of RGCs exposed to IOP elevation, we used a version of the microbead model of ocular hypertension to induce IOP elevation for 2 weeks^8^. At the end of the 2 week period, eyes were stratified into two groups, Mild IOP and Moderate IOP, which were defined according to strict criteria (see Methods). Normal IOP values for eyes (n = 30) of all three treatment groups in this study were 8.92 ± 0.26 and 9.1 ± 0.26 mmHg for right and left eye, respectively (Supplementary Fig. S1). In both the Mild and Moderate IOP groups, the change in IOP over the 2 weeks was constant and linear (Fig. 2a) with a significant average IOP increase of 2.7 ± 0.3 mmHg (P < 0.0001) and 7.0 ± 0.8 mmHg (P < 0.0001) for the Mild and Moderate IOP groups, respectively (Fig. 2b). Eyes injected with saline (Control) had no statistical increase in IOP over the 2 week period (Fig. 2a,b; average IOP difference = 0.0 ± 0.9 mmHg). After 2 weeks, RGCs were obtained as above from all samples, mRNA was isolated and pooled, and mRNA sequencing performed on technical replicates (see Methods). As an initial assessment of the effects of the two IOP elevations on RGC gene expression, a scatter plot based on principal component analysis (PCA, Fig. 2c) of the transcriptomic profile of the technical replicates was performed. PCA revealed that replicates from each treatment group clustered closely, and that there was clear separation among the Control, Mild IOP, and Moderate IOP treatment groups. Expression profiles of genes with an FPKM value >16 were then clustered using an unsupervised hierarchical approach. Again, treatment groups separated as expected (Control, Mild IOP, and Moderate IOP; Fig. 2d). Additional purity controls were also performed at this stage to further ensure that the analyzed population consisted primarily of RGCs (Supplementary Fig. S2).

### IOP Levels Produce Distinct RGC Transcriptomic Profiles

**Figure 3:**
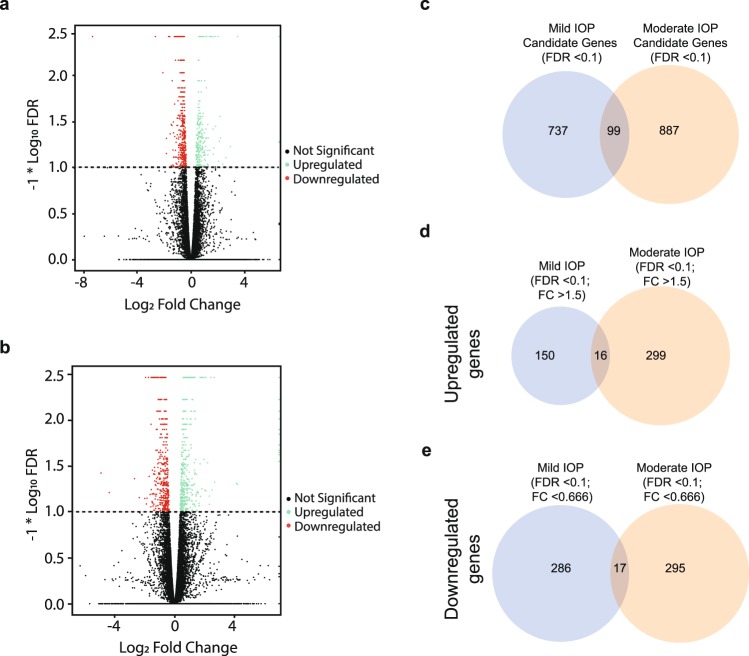
Differentially Expressed Genes Following Mild and Moderate IOP Elevation. (**a**,**b**) Volcano plots of significantly (FDR < 0.1) differently expressed genes (DEGs) for both Mild (**a**) and Moderate (**b**) IOP levels (above dotted line). Upregulated DEGs (Log_2_ fold change of ≥0.5849 or ≥1.5-fold change) and downregulated DEGs (Log_2_ fold change of ≤−0.5849 or ≤0.666-fold change), are indicated in green and red, respectively. Genes with Log_2_ Fold change of <|0.5849| are not significant (black). (**c**) Venn Diagram depicting the overlap of significant (FDR < 0.1) candidate DEGs in the Mild and Moderate IOP groups (does not include fold-change criteria). (**d**,**e**) Venn Diagrams of overlapping significantly (FDR < 0.1 and Log_2_ fold change of ≥|0.5849|) identified for up- (**d**) and down- (**e**) regulated DEGs.

We next sought to determine RGC differentially expressed genes (DEGs) following IOP elevation. We visualized these using volcano plots comparing the Mild (Fig. 3a) and Moderate (Fig. 3b) IOP groups to the control group. A total of 737 and 887 DEGs were considered significant (FDR < 0.1) candidates for the Mild and Moderate IOP groups, respectively (Fig. 3c). When fold-change (FC) criteria were additionally applied, a total of 449 DEGs were classified as significantly (FDR < 0.1; FC > 1.5) upregulated (Mild IOP = 150 DEGs; Moderate IOP = 299 DEGs; Fig. 3d). 581 DEGs were significantly (FDR < 0.1; FC < 0.666) downregulated (Mild IOP = 286 DEGs; Moderate IOP = 295 DEGs (Fig. 3e). The five most highly up- and down-regulated genes in the Mild and Moderate IOP groups are listed in Tables 1 and 2, respectively. Interestingly, there was very little overlap between the Mild and Moderate IOP groups, with only 16 commonly upregulated DEGs and 17 commonly downregulated DEGs detected (Fig. 3d,e; Supplementary Table S1). Additionally, only three DEGs were identified with regulation in the opposite direction according to IOP level (Supplementary Table S2). To confirm the RNA sequencing results, a subset of DEGs was selected for quantitative PCR. Critically, the fold changes of each tested gene paralleled the RNA sequencing data in response to Mild and Moderate IOP elevation (Table 3). To further confirm the validity of our RNA sequencing data, additional animals with elevated IOP were generated for all treatment groups (biological replicates), RGCs removed, and RNA isolated. When compared to our original RNA sequencing templates, these biological replicates showed similar significant qPCR expression trends (Supplementary Fig. S3).

**Table 1:** Top Up- and Down-regulated Genes Following Mild Elevation of IOP.

Gene Symbol	Gene Name	Gene Accession	Control FPKM Value	Mild IOP FPKM Value	Log_2_ Fold-Change	Adjusted P-value
**Upregulated**						
*Hist3h2bb-ps*	histone cluster 3 H2B family member b	NM_206882	0.00	2.84	∞	2.15E-03
*Tsacc*	TSSK6 activating cochaperone	NM_029801	0.00	2.63	∞	2.20E-03
*Gm20257*	caspase 8 pseudogene	NR_045007	0.00	1.89	∞	5.00E-05
*Mterf1b*	mitochondrial transcription termination factor 1	NM_001042670	0.00	1.81	∞	5.00E-05
*Car4*	carbonic anhydrase 4	NM_007607	0.00	1.77	∞	5.00E-05
**Downregulated**						
*Crygs*	crystallin gamma S	NM_009967	85.86	0.00	−∞	5.00E-05
*Cryba1*	crystallin beta A1	NM_009965	73.73	0.00	−∞	5.00E-05
*Crygb*	crystallin gamma B	NM_144761	18.35	0.00	−∞	5.00E-05
*Crygc*	crystallin gamma C	NM_007775	14.24	0.00	−∞	5.00E-05
*Gm4013*	Predicted gene 4013	NR_033452	4.83	0.00	−∞	7.00E-04

**Table 2:** Top Up- and Down-regulated Genes Following Moderate Elevation of IOP.

Gene Symbol	Gene Name	Gene Accession	Control FPKM Value	Moderate IOP FPKM Value	Log_2_ Fold-Change	Adjusted P-value
**Upregulated**						
*Vaultrc5*	vault RNA component 5	NR_027885	0.00	8.87	∞	2.15E-03
*Hist2h4*	histone cluster 1 H4 family member c	NM_033596	0.00	6.57	∞	2.20E-03
*Med9os*	mediator complex subunit 9, opposite strand	NR_045273	0.00	6.26	∞	5.00E-05
*Cd59b*	CD59 molecule (CD59 blood group)	NM_181858	0.00	4.94	∞	5.00E-05
*1700084E18Rik*	RIKEN cDNA 1700084E18 gene	NR_028299	0.00	4.43	∞	5.00E-05
**Downregulated**						
*Cryaa*	crystallin alpha A	NM_013501	127.75	0.00	−∞	1.40E-03
*Crygs*	crystallin gamma S	NM_009967	83.66	0.00	−∞	5.00E-05
*Cryba1*	crystallin beta A1	NM_009965	71.76	0.00	−∞	5.00E-05
*Cryba2*	crystallin beta A2	NM_021541	27.87	0.00	−∞	5.00E-05
*Crygb*	crystallin gamma B	NM_144761	17.81	0.00	−∞	5.00E-05

**Table 3:** RNA Sequencing Validation Through qPCR.

Genes	RNA Sequencing Data		qPCR Validation		
Gene Symbol	Log_2_ Fold-Change	P-value	Log_2_ Fold-Change ± SEM	P-value	*P < 0.05 **P < 0.01
**Mild IOP**					
*Tsacc*	∞	2.20E-03	5.7 ± 1.6	8.38E-02	
*Cp*	2.1	5.05E-03	1.1 ± 0.3	3.01E-02	*
*Trf*	1.1	2.60E-03	0.7 ± 0.2	3.97E-02	*
*Crygs*	−∞	5.00E-05	−8.7 ± 1.9	1.18E-02	*
*Cryba1*	−∞	5.00E-05	−9.2 ± 1.0	1.20E-02	*
*Cryaa/Cryaa2*	−7.3	5.00E-05	−7.2 ± 1.8	2.89E-02	*
**Moderate IOP**					
*Cp*	2.5	3.00E-03	2.1 ± 0.6	7.40E-03	**
*Trf*	2.4	5.00E-05	1.4 ± 0.3	1.60E-03	**
*Cryaa/Cryaa2*	−∞	5.00E-05	−8.6 ± 3.6	5.81E-02	
*Cryba1*	−∞	5.00E-05	−10.1 ± 1.2	2.78E-02	*
*Nab2*	−1.9	5.00E-05	−0.9 ± 0.2	3.90E-03	**

### Pathway Analysis in Response to Mild and Moderate IOP Levels

**Figure 4:**
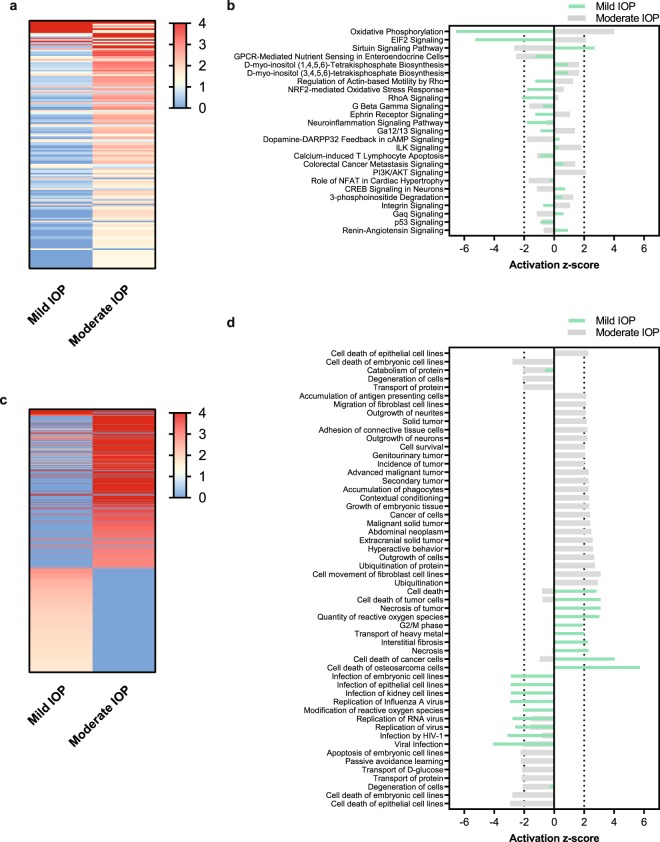
Pathway Analysis Following Mild and Moderate IOP. Ingenuity Pathway Analysis of Mild and Moderate IOP compared to Control. (**a**,**c**) Heatmap of the significant changes to the canonical pathway (**a**) and disease/bio functions (**c**) filtered by −log (P-value). (**b**,**d**) Activation z-score directionality graph of the top significant (−log (P-value) > 1.3) canonical pathways (**b**) and disease/bio functions (**d**) for both Mild and Moderate IOP elevation. Canonical pathways and disease/bio functions with a Z-score ≥ |2| are significant, were a positive value is considered to be activated and a negative value is considered to be inactivated.

We used Ingenuity Pathway Analysis (IPA; QIAGEN) to characterize alterations to RGC molecular signaling environments following Mild and Moderate IOP elevation. We initially used the Canonical Pathways feature in IPA and found that at both IOP levels, oxidative phosphorylation and mitochondrial dysfunction were the most highly regulated canonical pathways (Supplementary Table S3). Interestingly, the changes in these pathways for Mild and Moderate IOP elevation occurred in the opposite direction. For example, all of the detected genes involved in the oxidative phosphorylation pathway in the Mild IOP group were downregulated (44/109), whereas the genes in the Moderate IOP group were upregulated (16/109). Indeed, this finding of activation in opposite directions by Mild and Moderate IOP level was a common finding among the top 25 impacted canonical pathways (with a reported Z score), suggesting that Mild and Moderate IOP levels result in distinct and opposed transcriptional environments (Fig. 4a,b). We extended this analysis using the Disease and Bio Functions feature in IPA (Fig. 4c,d; Supplementary Tables S4 and S5) and obtained similar results - the Mild and Moderate IOP groups appear to exist in opposing molecular activation states. These results suggest that IOP elevation does not result in a static or linear injury but instead causes dynamic changes that are dependent on IOP and may have important thresholds.

## Discussion

Glaucoma is a multifactorial disease in which IOP, the major risk factor, impacts RGCs in a variety of ways according to the magnitude and duration of IOP exposure^46,47^. In this manuscript, we focused on IOP level and stratified adult mice according to Mild (1–4 mmHg) and Moderate (≥4 mmHg) IOP levels while maintaining a stable duration of IOP exposure (2 weeks). RNA sequencing of a pure population of immunopanned RGCs at each IOP level identified two distinct transcriptomic profiles, with only 33 out of 1,030 significant DEGs found to be common to both Mild and Moderate IOP groups. Further analysis of alterations of the two groups using the IPA canonical pathways and disease and bio functions were similarly distinct and showed an opposite directionality to their activation states. Taken together, these results suggest not only that the magnitude of IOP elevation has a critical impact on the RGC transcriptional environment, but that some IOP-based set point may exist within RGCs to impact the direction of transcriptional change.

Understanding the role of elevated IOP on the transcriptomic environment of adult RGCs has been of great interest for some time^31,37,38,48^. However, the determination of these adult RGC transcriptomic profiles has been hindered by an inability to isolate pure RGCs in large quantities despite protocols involving fluorescence-activated cell sorting (FACS), laser capture, and other forms of immunopanning^41,49–52^. In this study, we modified a well-established immunopanning technique to remove amacrine cells and thereby capture an extremely pure population of viable adult mouse RGCs. By avoiding FACS, we also minimized hydrodynamic stress and mechanical shearing^53^. Until recently^31^, the study of adult RGC transcriptomic profiles after IOP elevation has also been limited largely to microarray studies, both of which allow for detection only of a limited number of pre-determined transcripts^34,37,48,54^. The advantages of RNA sequencing over these approaches include higher sensitivity and dynamic range, which allows for a more comprehensive transcriptome^55^. By applying RNA sequencing techniques to RGCs isolated from retinas exposed to two IOP levels, we were able to profile distinct RGC transcriptomes according to IOP level. Importantly, we were able to validate this new and unexpected finding with independent biological replicates, suggesting that our results are not due to a spurious occurrence.

How do RGC transcriptomes differ according to IOP level? As the first study to describe and contrast the transcriptome, molecular pathway activation states, and disease/bio functions at more than one level of IOP elevation, we are able to begin to address this question. We found surprisingly little overlap in the DEGs identified between the two IOP levels, as well as a concomitant lack of overlap in molecular pathway activation states and disease/bio functions. Statistically significant changes to canonical pathways were observed in both IOP groups, most prominently impacting oxidative phosphorylation, mitochondrial dysfunction, EIF2 signaling, and Sirtuin signaling, some of which have been identified in previous publications^31,56^. However, the use of two IOP levels enabled us to determine that these pathways were altered in opposite directions based on IOP level. Inhibition of the oxidative phosphorylation pathway and EIF2 signaling pathways should cause mitochondria dysfunction, endoplasmic reticulum stress, and apoptosis and these pathways were inhibited only in the Mild IOP group (and activated in the Moderate IOP group)^57–59^. The Sirtuin signaling pathway may also play a role in RGC survival, as retinal SIRT1 protein and mRNA expression decreases following retinal ischemic-reperfusion injury and the activation of SIRT1 attenuates RGC loss in experimental optic neuritis^56,60^. Signaling of this pathway was activated only in the Mild IOP group (and inhibited in the Moderate IOP group). Thus, under conditions of both Mild and Moderate IOP there appears to be competition between distinct pro- and anti-apoptotic pathways. Since the directions of the inhibition/activation change with IOP level, this may signal not just a change in how RGCs respond to IOP, but how IOP level shifts the dominant molecular cellular processes. Looking further, in the Mild IOP elevation group, the disease/bio functions analysis showed activation of categories relating to ischemic/oxidative stress induced neuronal death. Interestingly, there was a seemingly opposite effect on the RGC molecular environment in the Moderate IOP group, which showed an increased activation of cell survival and neurite outgrowth. This may indicate that higher IOPs impact axonal/dendritic structures preferentially, whereas lower IOPs impact RGC somas. These distinctions will be important to understand if we are to develop systems to both prevent RGC death to promote RGC regeneration and axonal outgrowth.

Why do RGC transcriptomes differ according to IOP level? One potential interpretation of these results is that the changes seen after Mild IOP are the initial responses of RGCs, whereas the changes seen after Moderate IOP are the late responses. These later responses may be dominated by “healthier” or “modified” RGCs which survive the initial IOP insult. Said differently, the RGCs isolated after Mild IOP may represent all RGCs, whereas RGCs isolated after Moderate IOP may represent an IOP-resistant group in which the less resistant RGCs have already succumbed to the effects of IOP. While this is possible, it would be better studied by maintaining a constant IOP over a longer period and assessing various time points. Furthermore, as we obtained similar RGC yields at both IOP levels, it is unlikely that the RGCs isolated at the Moderate IOP level are solely “survivors.” Another possibility is that all changes are occurring simultaneously, but there is a preference toward certain kinds of responses according to IOP level. The opposing apoptosis pathways seen at distinct IOP levels may support this interpretation. Perhaps most likely, though, is that a set point (graded or with multiple thresholds) is present within RGCs that mediates IOP transcriptional responses. Accordingly, IOP may provoke certain intra- and extracellular signaling mechanisms unique to the different pressure levels. As there are many subtypes of RGCs, and these subtypes show differing functional responses in the presence of elevated IOP^29,61–64^, it is possible that part of what we see in this study represent the different transcriptional responses of these RGC subtypes.

How are RGC transcriptomes similar according to IOP level? Of the very few commonly regulated DEGs identified between the two IOP levels, most prominent were the downregulated genes for crystallins (Supplementary Table S1). Crystallins are found in RGCs, and optic nerve injury and rat models of glaucoma result in a decrease of transcription and translation of crystallins^65,66^. Overexpression of crystallins also can have a protective effect on RGCs during optic nerve injury^65^. Their identification in this study suggests that crystallins may have an important universal role in RGC injury. Some upregulated DEGs, such as ceruloplasmin and transferrin, are acute phase reactants and may simply signify an underlying stressed state. Other DEGs may represent important transcripts which are core IOP-based response genes – additional studies will be needed to make this determination.

Future studies on this topic are very likely to occur and will yield additional information on both differences and similarities in transcription according to IOP level. Studies on single RGCs might provide a clearer understanding of how individual subtypes of RGCs or retinal regions are affected by IOP elevation. Additionally, increasing the read depth of the RNA transcriptome could provide a more comprehensive transcriptomic profile, whether at the bulk or single cell level. Further studies with multiple IOP levels and multiple IOP exposure durations may help clarify the definition of these different transcriptomic environments. Lastly, proteomics analyses will help identify which of these critical transcriptional changes lead to functional change and may be the object of pharmacologic intervention for the treatment of glaucoma.

## Methods

### Ocular Hypertension/Experimental Glaucoma

All protocols and procedures were approved by the Institutional Animal Care and Use Committee of Baylor College of Medicine and conducted in accordance with the ARVO statement for the use of animals in ophthalmic and vision research as well as the United States Public Health Service’s Policy on Humane Care and the Use of Laboratory Animals. Female and male C57Bl/6J mice were purchased from Jackson Laboratories (Bar Harbor, ME, USA), and at six weeks of age, the procedure to elevate IOP was performed as previously published^8,9,28^. Based on weight, mice were initially anesthetized with an intraperitoneal injection of a combination solution of ketamine (80 mg/kg), xylazine (16 mg/kg), and acepromazine (1.2 mg/kg). The left eyes were given single drops of 1% tropicamide, 2.5% of phenylephrine hydrochloride and 0.5% proparacaine hydrochloride to dilate and locally anesthetized the eye. A 30-gauge needle was used to create an initial opening in the cornea. A pulled glass micropipette tip (~75 µm inner diameter) connected to a Hamilton syringe was used to inject a volume of 1.5–2 µL of treatment solution into the anterior chamber followed by 2 µL of sodium hyaluronate (#8065183085, Provisc; Alcon Laboratories, Fort Worth, TX, USA). Sodium hyaluronate was used to help guide the treatment solutions towards the iridocorneal angle. Treatment solutions consisted of either a combination of 6 µm (Cat#15715-5) and 1 µm (Cat# 15713-15) in diameter polystyrene microbeads (Polysciences, Inc., Warrington, PA, USA) or phosphate buffer saline (PBS), which either were the elevation in IOP injections or the control (vehicle) injections, respectively.

### Measurements of Intraocular Pressure

Mice were anesthetized using isoflurane and the IOPs of both eyes were observed with a rebound tonometer (#J1000Tl, Tonolab, ICare, Espoo, Finland). Baseline IOP measurements were taken before eyes were injected with either microbeads or saline. After the eye injection procedure, IOP measurements were taken three times a week at the same time of the day for a total duration of 2 weeks. IOP measurements from the injected eye were compared to the IOP measurements of the contralateral non-injected eyes to determine changes in IOP. Microbead injected eyes with an average IOP increase of either >1 and <4 mmHg or ≥4 mmHg were divided into mild IOP and moderate IOP groups, respectively. Saline-injected eyes served as controls with IOP change <±1 mmHg. Mice were carefully observed, where mice were removed from the study if there were signs of inflammation, opaque lens, or sporadic spikes (>30 mmHg) in IOP elevation. Significance for the cumulative change in IOP between the different IOP groups was determined using a 2-way ANOVA followed by the Dunnett’s multiple comparison test (**P < 0.01; ****P < 0.0001). A one-way ANOVA was performed using Tukey test to determine significance between the average IOP change per group (*P < 0.05; **P < 0.01; ****P < 0.0001).

### Immunopanning of Adult Mouse RGCs

RGCs were purified utilizing a 4-step immunopanning technique with an antibody against Thy1.2., modified from Barres *et al*.^41^. Actinomycin D [1 µg/mL] (#A7592, Life Technologies, Eugene, OR, USA) was supplemented into the Dulbecco’s Phosphate-Buffered Saline (DPBS) (#14287072, Life Technologies, Eugene, OR, USA) solutions used throughout the isolation preparation to inhibit new RNA transcription that would occur during the RGC isolation process. Retinas from injected eyes that fit in the inclusion conditions for control, Mild IOP, and Moderate IOP were dissected and enzymatically dissociated with 9 units/mL of papain (LS003126, Worthington, Lakewood, NJ, USA) for 45 mins at 34 °C. Retinal dissociations were then performed by trituration and filtration through a 20 µm nylon mesh (#SCNY00020, EMDMillipore, Burlington, MA, USA) resulting in a retinal cell suspension. Retinal cell suspensions were transferred then incubated on a negative panning plate coated with unconjugated Griffonia (Bandeiraea) Simplicifolia Lectin 1 (BSL-1) (#L-1100, Vector Laboratories, Burlingame, CA, USA) as previously described^41^. Two additional negative panning steps were then added, using plates coated with purified mouse CD11b/c (#554859, BD Pharmingen, San Jose, CA, USA), and monoclonal mouse anti-HNK-1/N-CAM (CD57)(VC1.1 clone) (#C6680-100TST, Sigma Aldrich, St. Louis, MO, USA). Together, these negative panning steps removed microglia, macrophages, endothelial cells, fibroblasts, and Thy1+ amacrine cells. The remaining cells in the retinal cell suspension that did not bind the negative panning plates were then transferred on to the positive panning plate containing bound antibodies against Thy1.2 (#MCA02R, Bio-Rad Antibodies, Hercules, CA, USA), creating a 4-step technique. Cells from Thy1.2 positives plates were then washed 25 times with DPBS to remove unbound retinal cells. Attached Thy1.2 positive cells were then immediately lysed in 4 °C TRIzol Reagent (#15596018, Life Technologies, Eugene, OR, USA). Samples from each plate were transferred and snapped frozen in 2 mL microcentrifuge tubes. TRIzol lysed RGC samples were stored in −80 °C until total RNA isolation was performed.

### Total RNA isolation

Total RNA from RGCs samples were isolated using a TRIzol/spin column-based nucleic acid extraction kit (Direct-Zol) (#R2050, Zymo Research, Irvine, CA, USA) according to manufacturer’s protocol. Linear Acrylamide [5 µg] (#AM9520, Life Technologies, Eugene, OR, USA) was added to each lysed RGC samples to be a co-precipitant, facilitating in higher precipitation of nucleic acids. Linear Acrylamide does not affect downstream RNA applications. DNase1 was added to the RNA extraction kit to remove genomic DNA. Total RNA was eluted in 50 µL of nuclease-free water. Total RNA quality and yield were assessed with the 2100 Bioanalyzer instrument using the Agilent 6000 kit (#5067-1513, Agilent, Santa Clara, CA, USA). Samples with RIN values > 7 were utilized in the study. For each treatment group [Control (n = 12), Mild IOP (n = 12), and Moderate IOP (n = 6)], 300 pg of total RNA per treated retina were pooled into a common sample and then split into triplicate technical replicates for sequencing.

### Quantitative Real-Time PCR

Intercalating dye primers targeting specific genes of interest were purchased from Integrated DNA Technologies, PrimeTime Predesigned qPCR assays or designed by NCBI Primer-BLAST. cDNA construction and the determination of quantitative expression of genes of interest (Supplementary Table S6) were performed using the iTaq Universal SYBR Green One-Step Kit (#1725151, Bio-Rad, Hercules, CA, USA). Conditions for qPCR reactions were followed accordingly to the manufacturer’s recommendation. qPCR reactions were run in triplicates and averaged. Housekeeping genes, *Hprt*, *Tubb5*, and *Ppia*, ran alongside our genes of interest, were *Hprt* was the most stable internal control and used for normalizing our genes of interest. Fold gene expression value were determined with the comparative C_t_ method (2^−ΔΔCt^). To determine statistical significance of *Rho* and *Pou4f2* in our isolated RGC samples compared to whole retinal sample, a 2-way ANOVA using the Sidak’s multiple comparison test was utilized (****P < 0.0001) A one-tailed unpaired t-test was performed to determine the significance of the genes of interest to validate the RNA sequencing (*P < 0.05; **P < 0.01; ****P < 0.0001).

### Immunocytochemistry and RGC Characterization

Immunocytochemistry was performed from 5 different RGC isolation preparations. A total of 10 mice (5 males and 5 females) were used. Two mice were utilized in each immunocytochemistry experiment. 50,000 RGCs were seeded onto each glass coverslips (#1254582, Fisher Scientific, Hampton, NH, USA) coated with poly-D-lysine (#P6407, Sigma-Aldrich, St. Louis, MO, USA). Cells were immediately fixed in 4% PFA for 15 mins at room temperatures and then permeabilized with 0.1% Triton X-100 for 5 mins. Blocking with 5% BSA and 5% normal donkey serum was performed for 1 hour at room temperature and then cells were incubated with rabbit anti-RBPMS (1:250 dilution, #1830-RBPMS, PhosphoSolutions, Aurora, CO, USA) and mouse anti-TUJ1 (1:300 dilution, #MMS-435P, San Diego, CA, USA) antibodies overnight in a moist chamber at 4 °C. The next morning, cells were then incubated in appropriated secondary Alexa Fluor conjugated antibodies (1:1000 dilution, #A21207, #A21203, Thermo Fisher Scientific, Waltham, MA, USA) for 1 hour at room temperature. Prolong Diamond antifade with DAPI (#P36971, Thermo Fisher Scientific, Waltham, MA, USA) was then applied to slides where coverslips were then placed on. Images were taken on a Leica DMi8 inverted microscope (Buffalo Grove, IL, USA). A total of six images were acquired per coverslip at x10 magnification using a fixed 3 × 2 grid. The diameters in each individual cell were measured in each image. The cell size distribution was obtained using ImageJ (NIH) to measure cell diameter from brightfield images. Counting of the RBPMS staining was performed in a masked manner where RBPMS staining was compared to DAPI staining to determine a positive cell. Viability was determined immediately after the trypsinization of the cells following the 4-step immunopanning technique. Dead cells were stained with Trypan Blue (0.4%; #15250061, Gibco, Waltham, MA, USA) and viability was determined with the Countess Cell Counter (#C10281, Invitrogen, Carlsbad, CA, USA).

### Library Preparation and RNA Sequencing

The RNA sequencing libraries were generated according to SMART-seq v4 Ultra low input RNA kit (#634888, Takara Clontech, Mountain View, CA, USA). In brief, purified RNA was incubated with lysis buffer for 5 minutes, 3-SMART-seq CDS primer II (modified Oligo-dT primer) and V4 oligonucleotide were added to isolated RNA for first stranded cDNA synthesis. cDNA was amplified using PCR Primer II A, and subsequently purified using Ampure XP beads (#A63880, Beckman Coulter Life Science, Indianapolis, IN, USA). The Illumina library was prepared using Nextera XT DNA library preparation kit (#FC-131–1024, Illumina, San Diego, CA, USA), and sequenced using Illumina HiSeq2500 using 2 × 100 bp flow cell for 20 million paired-end reads per sample. Library preparation and sequencing was performed at the Human Genome Sequencing Center (Baylor College of Medicine, Houston, TX).

### Differential Gene Expression (DEGs) and Pathway Analysis

Reads were aligned to the mm10 *Mus musculus* genome assembly and transcriptome using a bowtie2 (v2.2.3), cufflinks (v2.2.1), and cuffdiff pipeline^67–69^ on the MD Anderson High Powered Computing core facility. Data were processed using R (v3.4.1) using the Bioconductor libraries (v3.7) cummeRbund and DEseq2. Cuffdiff Q-value (False Discovery Rate (FDR) corrected P-value) was set at 0.1 for the differential expression analysis. Genes with an FDR < 0.1 along with Log_2_ fold changes ≥|0.5849| (≥0.5849 = Fold Change (FC) > 1.5; ≤0.549 = FC < 0.666) were considered to be significantly up- or downregulated differently expressed genes (DEGs). Pathway analysis and disease and biology functions were determined by QIAGEN’s Ingenuity Pathway Analysis (IPA, QIAGEN, Germantown, MD, USA). The list of genes (genes with FDR < 0.1) from the Mild and Moderate IOP groups were inputted into IPA. Significant canonical pathways, disease and biology functions were determined with threshold set at −log(P-value) > 1.3 and/or activation z-score of ≥|2|.

### Statistical Analysis

GraphPad Prism 7 (La Jolla, CA, USA) was used to perform our statistical analysis. One-tailed t-test, 1-way, and 2-way ANOVAs were performed with appropriate post hoc test as stated in the methods above. Statistical significance of the experimental data was described as *P < 0.05; **P < 0.01; ***P < 0.001; ****P < 0.0001. Data are presented as mean ± SEM.

## Supplementary information

Supplementary Information

## Data Availability

The data discussed in this publication have been deposited in NCBI’s Gene Expression Omnibus and are accessible through GEO Series accession number GSE122205.
