# Supplementary material for: Transcriptomic profiles of retinal ganglion cells are defined by the magnitude of intraocular pressure elevation in adult mice
Source: Sci Rep. 2019 Feb 22;9:2594. doi: 10.1038/s41598-019-39141-1 (PMC6385489; doi:10.1038/s41598-019-39141-1)
Supplement: Supplementary file 1 — Supplementary Information [file 41598_2019_39141_MOESM1_ESM.pdf]

Supplementary Information

Title: Transcriptomic profiles of retinal ganglion cells are defined by the magnitude of intraocular pressure elevation in adult mice

Authors: Yong H. Park, Joshua D. Snook, Edwin J. Ostrin, Sangbae Kim, Rui Chen, Benjamin J. Frankfort

Supplementary Figures S1 to S3

Supplementary Tables S1 to S6

Supplementary Figure S1

a

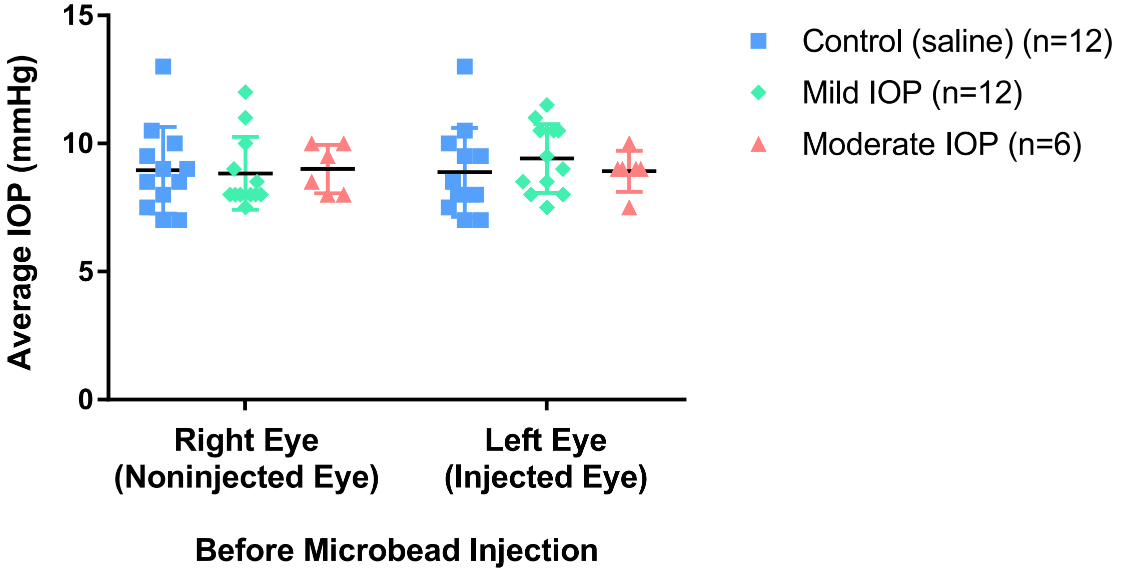

b

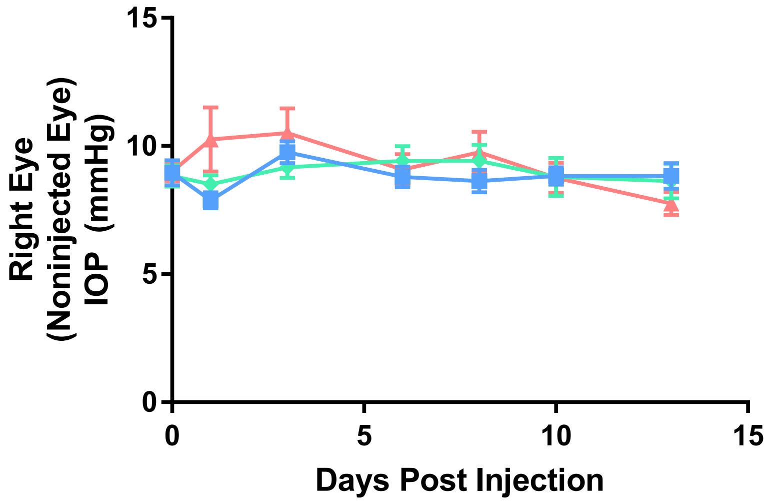

Figure S1. IOP measurements before and after microbead injection

IOP measurements of both right (noninjected) and left (injected) eye before microbead injection for all treatment groups (a). Average IOP measurements of right (noninjected) eye for all treatment groups for a period of 2 weeks following injection (b).

Supplementary Figure S2

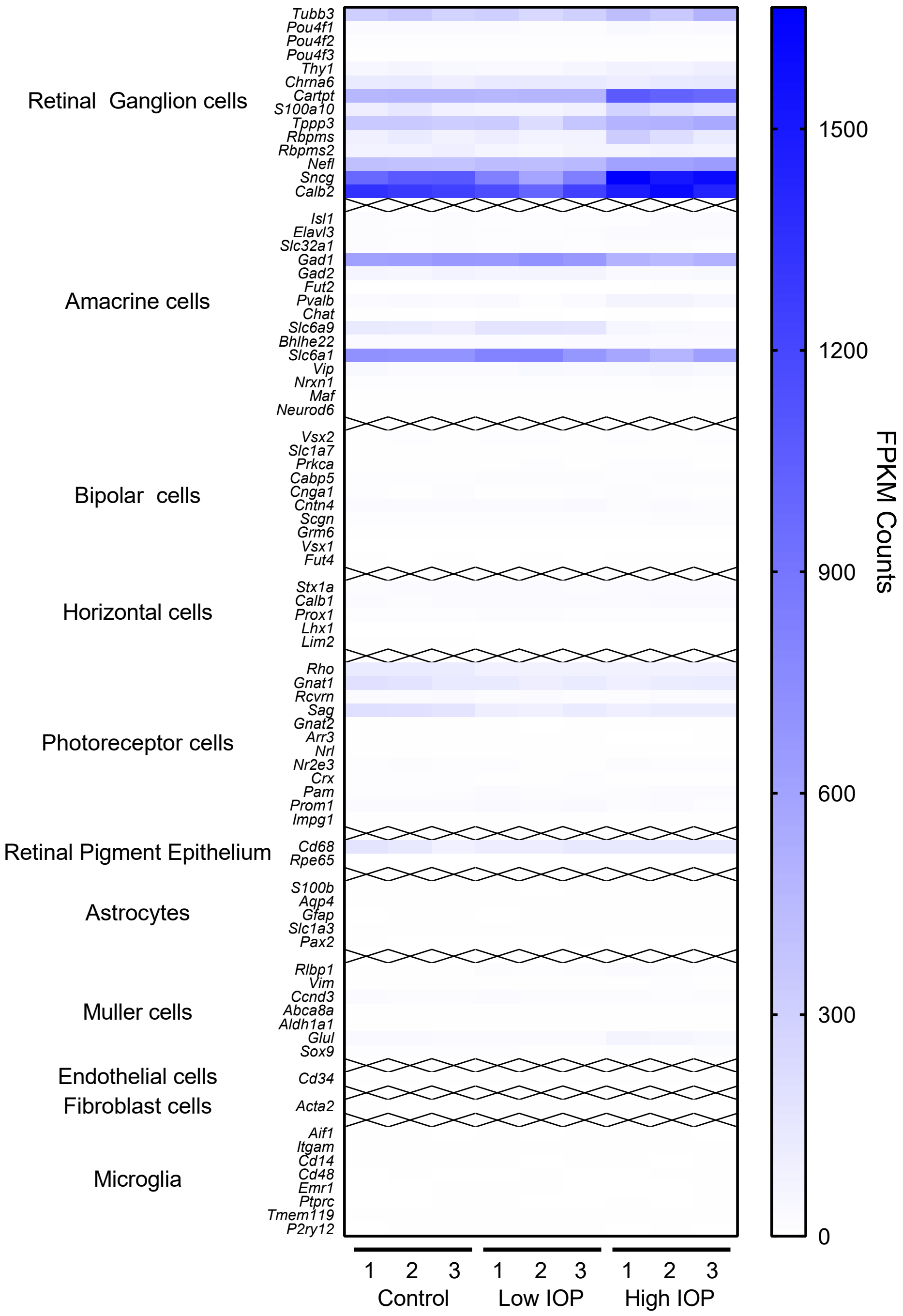

Figure S2. Confirmation of RGC purity in samples through RNA Sequencing

Transcriptome profile of each RNA sample was identified through RNA sequencing and normalized by FPKM. Retinal cell marker FPKM expression counts were compared among treatment groups. High expression of expected RGC markers was consistently detected, with minimal expression of markers for other retinal cell types.

Supplementary Figure S3

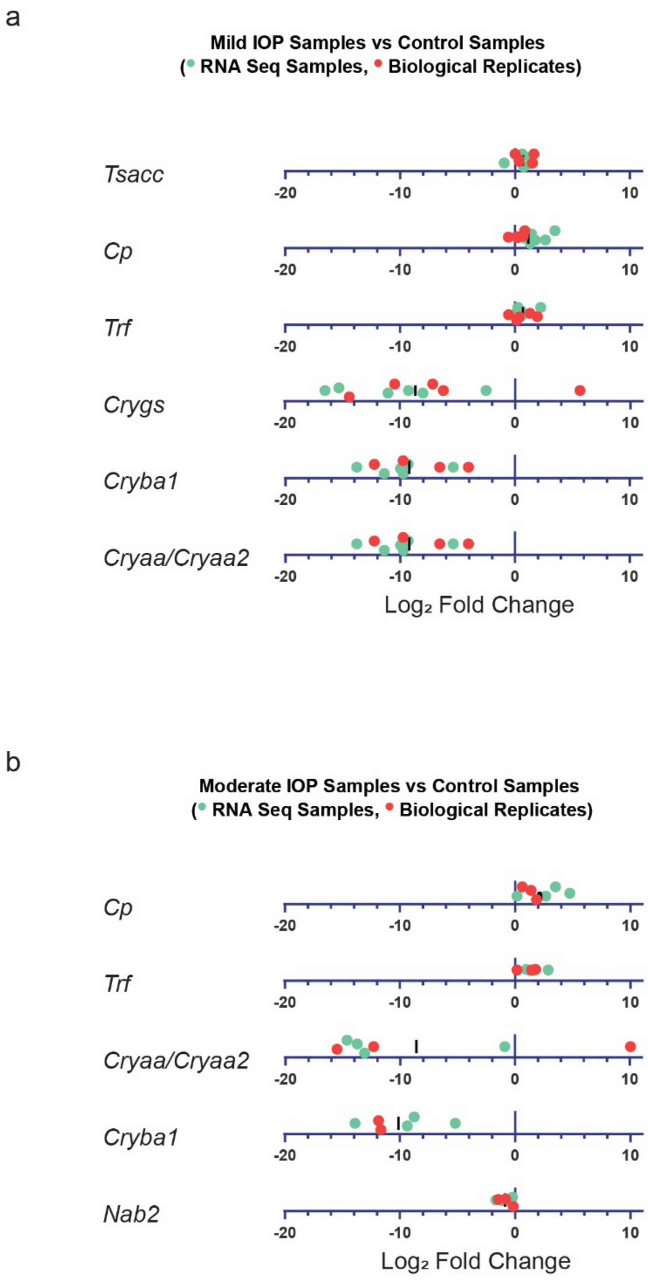

Figure S3. qPCR validation of Mild and Moderate IOP DEGs Identified through RNA Sequencing

Validation of selected DEGs from Mild (a) and Moderate (b) IOP groups were confirmed by qPCR using RNA sequencing samples and a new cohort of biological samples. Graphical Log_2 Fold Change expression of selected DEGs from initial RNA sequencing samples (green) and confirmatory biological replicates (red) compared to controls.

Gene Symbol	Gene Name	Gene Accession	Mild IOP Log_2 Fold-Change	Moderate IOP Log_2 Fold-Change
**Upregulated**				
Gm20257	caspase 8 pseudogene	NR_045007	∞	∞
Cp	ceruloplasmin	NM_007752	2.07	2.50
Ubc	ubiquitin C	NM_019639	1.62	1.30
Gabrr2	gamma-aminobutyric acid type A receptor rho2 subunit	NM_008076	1.15	1.08
Trf	transferrin	NM_133977	1.10	2.41
Cyb561	cytochrome b561	NM_007805	0.84	1.13
C1orf216	chromosome 1 open reading frame 216	NM_001145950	0.84	1.36
Gnpda2	glucosamine-6-phosphate deaminase 2	NM_001038015	0.83	0.70
Kif2a	kinesin family member 2A	NM_008442	0.80	1.16
Ophn1	oligophrenin 1	NM_052976	0.72	0.82
Them4	thioesterase superfamily member 4	NM_029431	0.70	0.69
Tigd2	tigger transposable element derived 2	NM_001081145	0.69	1.05
Usp1	ubiquitin specific peptidase 1	NM_146144	0.66	0.66
Tet1	tet methylcytosine dioxygenase 1	NM_027384	0.66	0.78
Epha4	EPH receptor A4	NM_007936	0.66	1.00
Vipr2	vasoactive intestinal peptide receptor 2	NM_009511	0.64	0.74
**Downregulated**				
Cryba1	Crystallin Beta A1	NM_009965	-∞	-∞
Crygb	Crystallin Gamma B	NM_144761	-∞	-∞
Crygc	Crystallin Gamma C	NM_007775	-∞	-∞
Crygs	Crystallin Gamma S	NM_009967	-∞	-∞
Cryaa/Cryaa2	Crystallin Alpha A	NM_013501	-7.34	-∞
Kcnu1	Potassium Calcium-Activated Channel Subfamily U Member 1	NM_008432	-1.30	-1.32
Gtf2h3	General Transcription Factor Iih Subunit 3	NM_181410	-0.95	-0.93
Phgdh	Phosphoglycerate Dehydrogenase	NM_016966	-0.88	-1.08
Tnfaip3	Tnf Alpha Induced Protein 3	NM_009397	-0.88	-1.24
Cacnb1	Calcium Voltage-Gated Channel Auxiliary Subunit Beta 1	NM_145121	-0.81	-1.05
Bcam	Basal Cell Adhesion Molecule	NM_020486	-0.80	-0.89
Kiaa1211l	Kiaa1211 Like	NM_028096	-0.68	-1.74
Stc1	Stanniocalcin 1	NM_009285	-0.66	-0.68
Cavin3	Caveolae Associated Protein 3	NM_028444	-0.63	-0.94
Rassf1	Ras Association Domain Family Member 1	NM_019713	-0.63	-0.69
Tob2	Transducer Of Erbb2, 2	NM_020507	-0.62	-0.72
Tgfb3	Transforming Growth Factor Beta 3	NM_009368	-0.61	-0.87

Supplementary Table S1. DEGs Regulated in the Same Direction After Both Mild and Moderate Elevation of IOP

Gene Symbol	Gene Name	Gene Accession	Mild IOP Log_2 Fold-Change	Moderate IOP Log_2 Fold-Change
Txn1	thioredoxin	NM_011660	-0.70	0.61
Fxyd7	FXYD domain containing ion transport regulator 7	NM_022007	-0.78	0.86
Ebpl	emopamil binding protein like	NM_026598	-0.79	0.81

Supplementary Table S2. DEGs Regulated in the Opposite Direction After Both Mild and Moderate Elevation of IOP

Canonical Pathways	-log(P-value)	# of Genes Overlap with IPA Data Set	Upregulated Genes Overlap with IPA Data Set	Downregulated Genes Overlap with IPA Data Set
**Mild IOP**				
Oxidative Phosphorylation	36.5	44/109	0/109 (0%)	44/109 (40%)
Mitochondrial Dysfunction	32.4	49/171	0/171 (0%)	49/171 (29%)
EIF2 Signaling	31.8	54/227	1/221 (0%)	53/221 (24%)
Sirtuin Signaling Pathway	12.1	37/292	1/292 (0%)	36/292 (12%)
Regulation of eIF4 and p70S6K Signaling	8.3	22/163	1/157 (1%)	21/157 (13%)
**Moderate IOP**				
Mitochondrial Dysfunction	5.6	21/171	18/171 (11%)	3/171 (2%)
Oxidative Phosphorylation	5.4	16/109	16/109 (15%)	0/109 (0%)
NRF2-mediated Oxidative Stress Response	4.7	22/199	14/199 (7%)	8/199 (4%)
14-3-3-mediated Signaling	4.6	17/137	9/137 (6%)	8/137 (6%)
Gap Junction Signaling	4.4	21/201	7/201 (4%)	14/201 (7%)

Supplementary Table S3. Top 5 Canonical Pathways Activated in Both Mild and Moderate Elevation of IOP

Categories	Genes (*n*)	P-value Range
**Diseases and Disorders**		
Cancer	155	1.83E-02 – 6.96E-34
Organismal Injury and Abnormalities	318	1.83E-02 – 6.96E-34
Tumor Morphology	75	1.79E-02 – 6.96E-34
Developmental Disorder	57	1.45E-02 – 2.16E-11
Hereditary Disorder	122	1.56E-02 – 2.16E-11
**Molecular and Cellular Functions**		
RNA Damage and Repair	58	2.43E-03 – 9.47E-43
Protein Synthesis	145	8.02E-03 – 5.90E-37
Cell Death and Survival	231	1.83E-02 – 6.96E-34
Cell Signaling	56	1.89E-02 – 1.42E-20
Post-Translational Modification	89	8.02E-03 – 1.42E-20
**Physiological System Development and Function**		
Behavior	44	1.30E-02 – 6.25E-05
Organismal Functions	34	1.34E-03 – 6.25E-05
Embryonic Development	34	1.83E-02 – 1.06E-04
Nervous System Development and Function	89	1.78E-02 – 1.06E-04
Organ Development	36	1.70E-02 – 1.06E-04

Supplementary Table S4. Top 5 Diseases and Bio Functions Identified in Mild Elevation of IOP

Categories	Genes (*n*)	P-value Range
**Diseases and Disorders**		
Neurological Disease	328	8.30E-04 – 6.79E-18
Skeletal and Muscular Disorder	112	3.12E-04 – 7.69E-18
Cancer	775	8.13E-04 – 3.03E-15
Organismal Injury and Abnormalities	790	8.30E-04 – 3.03E-15
Psychological Disorder	187	8.28E-04 – 3.66E-14
**Molecular and Cellular Functions**		
Cellular Assembly and Organization	236	7.78E-04 – 6.83E-17
Cellular Function and Maintenance	263	8.09E-04 – 6.83E-17
Cell Morphology	236	8.02E-04 – 7.48E-17
Cellular Development	296	7.28E-04 – 7.48E-17
Cellular Growth and Proliferation	266	7.28E-04 – 7.48E-17
**Physiological System Development and Function**		
Nervous System Development and Function	269	8.30E-04 – 7.48E-17
Organismal Functions	252	8.30E-04 – 7.48E-17
Tissue Development	238	7.00E-04 – 7.48E-17
Organismal Survival	246	8.12E-04 – 1.97E-13
Behavior	140	6.05E-04 – 1.28E-11

Supplementary Table S5. Top 5 Diseases and Bio Functions Identified in Moderate Elevation of IOP

#	Gene Target	Forward or Reverse Primers	Primer Sequence (5’-3’)	IDT PrimeTime® qPCR Primers Assay ID	Product Size (bp)
1	*Tsacc*	F’-<br>R’-	GAC CAT CCA GAC AAA GCT GT<br>AGC TAT CTG TCG TTG AGC AAG	Mm.PT.56a.32791036	121
2	*Cp*	F’-<br>R’-	GAC GGA CAT CTT CAC TGG AG<br>CTG TGT ACT CAC GAT ATG CCA T	Mm.PT.58.12453979	122
3	*Trf*	F’-<br>R’-	AGA TAG AGT GTG AGT CAG CAG A<br>TCT CGT AGT ACT CTG CCA TGA	Mm.PT.58.23794874	147
4	*Crygs*	F’-<br>R’-	CAA TGT CTA AAA CTG GAG GCA AG<br>TTA GGT ACG AGC GGA AGT CT	Mm.PT.58.10545508	105
5	*Cryba1*	F’-<br>R’-	GAG CAA TGC CTA TCA TAT TGA GC<br>CAC TGG CGT CCA ATA AAG TTC	Mm.PT.58.29082641	114
6	*Cryaa/Cryaa2*	F’-<br>R’-	TCT TGG ACG TGA AGC ACT T<br>CAT TGG AAG GCA GAC GGT AG	Mm.PT.58.41330088	150
7	*Nab2*	F’-<br>R’-	TGG CAG AGG GGA TAA CAC A<br>GTA GTA AGA GAG GAG GTT GGC	Mm.PT.58.9432256	136
8	*Pou4f2*	F’-<br>R’-	GCA GTC TCC ACT TGG TGC TTA C<br>TTC CCC CTA CAA ACA AAC CTC C		149
9	*Rho*	F’-<br>R’-	CGC ACA CCC CTC AAC TAC AT<br>CAG GGC GAT TTC ACC TCC AA		171
10	*Hprt*	F’-<br>R’-	TCA GTC AAC GGG GGA CAT AAA<br>GGG GCT GTA CTG CTT AAC CAG		142
11	*Ppia*	F’-<br>R’-	CAA ACA CAA ACG GTT CCC AG<br>TTC ACC TTC CCA AAG ACC AC	Mm.PT.39a.2.gs	85
12	*Tubb5*	F’-<br>R’-	TCT TGT TCG GTA CCT ACA TTG G<br>TCG CTT ATC ACC TCC CAG AA	Mm.PT.58.28837085	135

Supplementary Table S6. Primers for Quantitative Real-Time PCR
